# Parkinson's Disease DJ-1 L166P Alters rRNA Biogenesis by Exclusion of TTRAP from the Nucleolus and Sequestration into Cytoplasmic Aggregates via TRAF6

**DOI:** 10.1371/journal.pone.0035051

**Published:** 2012-04-20

**Authors:** Sandra Vilotti, Marta Codrich, Marco Dal Ferro, Milena Pinto, Isidro Ferrer, Licio Collavin, Stefano Gustincich, Silvia Zucchelli

**Affiliations:** 1 SISSA, Sector of Neurobiology, Trieste, Italy; 2 Laboratorio Nazionale Consorzio Interuniversitario Biotecnologie, Trieste, Italy; 3 Department of Life Sciences (DSV), University of Trieste, Trieste, Italy; 4 Institute of Neuropathology, Institut d'Investigacio Biomedica de Bellvitge, University Hospital Bellvitge, University of Barcellona, Llbregat, Spain; 5 SISSA Unit, Italian Institute of Technology (IIT), Trieste, Italy; University of Turin, Italy

## Abstract

Mutations in PARK7/DJ-1 gene are associated to autosomal recessive early onset forms of Parkinson's disease (PD). Although large gene deletions have been linked to a loss-of-function phenotype, the pathogenic mechanism of missense mutations is less clear. The L166P mutation causes misfolding of DJ-1 protein and its degradation. L166P protein may also accumulate into insoluble cytoplasmic aggregates with a mechanism facilitated by the E3 ligase TNF receptor associated factor 6 (TRAF6). Upon proteasome impairment L166P activates the JNK/p38 MAPK apoptotic pathway by its interaction with TRAF and TNF Receptor Associated Protein (TTRAP). When proteasome activity is blocked in the presence of wild-type DJ-1, TTRAP forms aggregates that are localized to the cytoplasm or associated to nucleolar cavities, where it is required for a correct rRNA biogenesis. In this study we show that in post-mortem brains of sporadic PD patients TTRAP is associated to the nucleolus and to Lewy Bodies, cytoplasmic aggregates considered the hallmark of the disease. In SH-SY5Y neuroblastoma cells, misfolded mutant DJ-1 L166P alters rRNA biogenesis inhibiting TTRAP localization to the nucleolus and enhancing its recruitment into cytoplasmic aggregates with a mechanism that depends in part on TRAF6 activity. This work suggests that TTRAP plays a role in the molecular mechanisms of both sporadic and familial PD. Furthermore, it unveils the existence of an interplay between cytoplasmic and nucleolar aggregates that impacts rRNA biogenesis and involves TRAF6.

## Introduction

The accumulation of misfolded proteins is a common feature to a wide range of neurodegenerative diseases [1]. A neuropathological hallmark of Parkinson's disease (PD) is the presence of proteinaceous aggregates, known as Lewy Bodies (LBs), in the cytoplasm of dopaminergic (DA) neurons of the Substantia Nigra, the major target of neurodegeneration. In Huntington's disease (HD), N-terminal fragments of mutant huntingtin (N-HTT) form intracellular aggregates in the brain [2].

Several evidences implicate malfunction of the ubiquitin-proteasome system (UPS) in both idiopatic and familial PD [3]. Key UPS elements are altered in PD post-mortem brains [4], while synthetic proteasome inhibitors preferentially affect catecholaminergic neurons *in vitro* and *in vivo* leading to cell death [5], [6].

DJ-1 is a ubiquitously expressed protein that is mutated in autosomal recessive early onset forms of PD (PARK7) [7]. DJ-1 is an anti-oxidant protein [8] involved in mitochondrial integrity [9], autophagy [10] and hypoxia [11], [12]. Together with phenotypes due to large gene deletions, the exact pathogenic mechanism of PD-causing missense mutations remains unclear. In the most studied case, L166P disrupts DJ-1 protein activity and dimer formation, resulting in a misfolded protein that undergoes degradation [13]. However, mutant DJ-1 may also accumulate into insoluble cytoplasmic aggregates [14], [15] and acquire a gain-of-function property that may co-exist with its loss of physiological activity, as previously found for mutant HTT in HD [16] and superoxide dismutase 1 (SOD1) missense mutations in amyotrophic lateral sclerosis (ALS) [17].

The formation of L166P-containing aggregates is facilitated by atypical ubiquitination carried out by the TNF associated protein 6 (TRAF6) [15], a component of LBs in PD post-mortem brains. Interestingly, TRAF6 activity also triggers aggregate formation of mutant HTT suggesting a broader role of this E3 ligase in neurodegenerative diseases [18].

TRAF and TNF Receptor Associated Protein (TTRAP) is a 5′-tyrosyl DNA phosphodiesterase that repairs topoisomerase-2-induced DNA breaks [19]. In physiological conditions in neurons TTRAP is a nuclear protein associated to PML Nuclear Bodies (PML-NBs) [20]. It was originally described as involved in signal transduction for its interaction with TNF receptors (TNFR) family members and TNFR-associated factors (TRAFs), including TRAF6 [21], as well as in transcriptional regulation for its binding to ETS-related proteins [22]. TTRAP has been linked to PD for its ability to specifically interact with L166P DJ-1. When UPS activity is inhibited, TTRAP mediates L166P toxicity via JNK/p38 MAPK pathways providing the molecular basis for L166P gain-of-function properties [23]. Interestingly, TTRAP forms aggregates both in the cytoplasm and in nucleolar cavities, a region of the nucleolus devoid of ribosomal markers [24]. Under these conditions, TTRAP is neuroprotective and required for a correct rRNA biogenesis [20].

The structure and function of the nucleolus have been recently found altered in PD and essential to the survival of DA neurons *in vivo* [25], [26].

In this paper we show that in PD post-mortem brains TTRAP is associated to LBs in the cytoplasm or accumulated in the nucleolus in a portion of surviving DA neurons. In a cellular model of familial PD linked to mutant DJ-1 L166P, accumulation of misfolded proteins impairs rRNA biogenesis *in vitro* by inhibiting TTRAP localization into nucleolar cavities. This is concomitant with an increase in the number and size of cytoplasmic aggregates containing both L166P and TTRAP. This phenotype depends, at least in part, on TRAF6 E3 ligase activity.

## Materials and Methods

### Human post-mortem brains

Brain samples were obtained from the brain bank at the Institute of Neuropathology, Bellvitge Hospital (University of Barcelona, Spain). Samples were dissected at autopsy with the informed consent of patients or their relatives and the institutional approval of the Ethics Committee of the University of Barcelona. Brains were obtained from Caucasian, pathologically confirmed PD cases and age-matched controls [27]. Briefly, all cases of PD had suffered from classical PD, none of them had cognitive impairment and their neuropathological characterization was made according to established criteria. Control healthy subjects showed absence of neurological symptoms and of metabolic and vascular diseases, and the neuropathological study disclosed no abnormalities, including lack of Alzheimer disease and related pathology. The time between death and tissue preparation was in the range of 3 to 5 hours. The ventral midbrain region was sectioned horizontally. The dark pigmented zones of the Substantia Nigra were readily apparent from all surrounding structures and were then isolated from the ventral midbrain. For histological analysis, samples were cryoprotected with 30% sucrose in 4% formaldehyde, frozen in dry ice and stored at −80°C until use.

### Immunohistochemistry

Immunohistochemistry was performed on three controls and six PD human post-mortem brains. Only Substantia Nigra was dissected and analyzed. Tissue processing and histology were performed as previously described [15]. The following primary antibodies were used: anti-TTRAP (1∶50) [23] and anti-alpha-synuclein (1∶100, Cell Signaling Technology). Nuclei were visualized with DAPI. DA neurons in the Substantia Nigra were identified by neuromelanin staining with transmitted light.

### Cell culture and transfections

Human neuroblastoma SH-SY5Y cells (*ATCC*) were maintained in culture as suggested by the vendor. SH-SY5Y cells stably transfected with pCDNA3-FLAG-DJ-1 wt and L166P or empty vector control and with pSuperior-siDJ-1 or pSuperior-Scramble were previously described [11], [23]. All stably transfected SH-SY5Y cells were kept in culture with neomycin selection. Proteasome inhibition was performed for 16 hours with 5 µM MG132 (z-Leu-Leu-al) (Sigma Aldrich). DJ-1 silencing was obtained by adding 2.5 µg/ml of Doxycicline Hyclate (Sigma Aldrich) every 48 hours for a total of 10 days. GFP-TRAF6 (wt and DN) and N-HTT-GFP Gln^21^ and Gln^150^ were previously described [15], [18]. Transfections were performed with Lipofectamine reagent (Invitrogen).

For silencing endogenous TRAF6 specific oligonucleotides (Sigma Aldrich, EHU147511) were transiently transfected with Oligofectamine (Invitrogen). Immunofluorescence and western blot experiments were performed 72 hours after transfection.

### Immunofluorescence

For immunofluorescence experiments, cells were fixed in 4% paraformaldehyde directly added to culture medium for 10 minutes, then washed in PBS two times, treated with 0.1 M glycine for 4 minutes in PBS and permeabilized with 0,1% Triton X-100 in PBS for another 4 minutes. After washing with PBS and blocking with 0.2% BSA, 1% NGS, 0,1% Triton X-100 in PBS (blocking solution), cells were incubated with the indicated antibodies diluted in blocking solution for 90 minutes at room temperature. To detect insoluble TTRAP, immunocytochemistry was performed as described earlier [23].

We used anti-FLAG (1∶1000, Sigma Aldrich), anti-TTRAP (1∶100), anti-NPM (1∶100, Invitrogen), anti-Nucleolin (1∶100, Invitrogen), anti-PML (PG-M3) (1∶100, Santa Cruz Biotechnology), anti-p53 (DO1, Santa Cruz Biotechnology). For detection, cells were incubated with Alexa Fluor-488 or -594 (Molecular Probes, Invitrogen) labeled anti-mouse or anti-rabbit secondary antibodies. For nuclear staining, cells were incubated with DAPI (1 µg/ml) for 5 minutes. Triple immunofluorescence was performed using Zenon technology: anti-NPM antibody was labeled with Zenon Alexa Fluor 405 Mouse IgG(1) labeling reagent (Invitrogen), according to manufacturer's instructions. Cells were washed and mounted with Vectashield mounting medium (Vector). All images were collected using a confocal laser scanning microscopy LEICA TCS SP2. The analysis of aggregates was performed on high-resolution images using ImageJ software. At least 100 cells from two independent experiments were counted and scored for percentage of cells with aggregates and average aggregate size.

### RNA isolation, reverse transcription and qPCR

Total RNA was isolated using the TRIZOL reagent (Invitrogen) following the manufacturer's instructions. Single strand cDNA was obtained from 1 µg of purified RNA using the iSCRIPT™ cDNA Synthesis Kit (Bio-Rad) according to manufacturer's instructions. Quantitative real time PCR (qPCR) was performed using SYBR-Green PCR Master Mix (Applied Biosystem) and an iCycler IQ Real time PCR System (Bio-Rad). Primers for rRNA biogenesis A0, 1 and 4 [20], and for housekeeping genes beta-actin and GAPDH [15] were previously described.

### ^32^P-orthophosphate in vivo labeling and RNA analysis

Metabolic labeling and analysis of rRNA was carried out as previously described [20]. Briefly, mutant DJ-1 L166P and empty control cells were treated with 5 µM MG132 for 16 h or left untreated. Drug was maintained in the medium during the whole time of the procedure. Cells were incubated with phosphate-free medium supplemented with 10% dialyzed FBS (labeling medium) for 1 h prior to labeling with ^32^P-orthophosphate (15 µCi/ml) in labeling medium for 1 h (pulse). Medium was then replaced with complete medium for 3 h (chase). Total RNA was extracted using TRIZOL reagent (Invitrogen) and 1 µg of purified RNA was separated on 1% agarose-formaldehyde gel. After electrophoresis, 28S and 18S rRNA forms were controlled under UV light and gels were dried. rRNA species were detected by autoradiography.

### Proliferation and viability assays

Cell proliferation was measured by flow-cytometry (FACS) upon propidium iodide staining. Briefly, SH-SY5Y cells stably transfected with L166P and control cells were either left untreated or treated with 5 µM MG132 for 16 hours. After treatment, cells were collected, fixed with ice-cold 70% ethanol, treated with RNase A (0.2 mg/ml), and stained with propidium iodide (0.04 mg/ml). Samples were analyzed on a flow cytometer (FacsCalibur, BD). FACS data were processed using FlowJo software, and cell cycle profiles were determined using the Watson pragmatic model (Tree Star).

Cell viability was measured using WST-1 reagent (Roche), following manufacturer's instructions and reading absorbance at 440 nm using a standard spectrophotometer. Percentage of live cells after treatment with 5 µM MG132 for 16 hours was calculated relative to DMSO-treated cells (set as 100% for each cell line).

### Cell fractionation and Western blot analysis

SH-SY5Y stable cells were lysed in a buffer containing 150 mM NaCl, 50 mM Tris pH 7.5 and 0.2% TRITON X-100, supplemented with protease inhibitor cocktail (Roche). Lysates were centrifuged at 20 000 g for 30 min at 4°C and separated into Triton X-100 soluble (supernatant) and insoluble (pellet) fractions. Insoluble pellets were resuspended in boiling sample buffer, sonicated and used for western blot analysis. The following antibodies were used: anti-FLAG 1∶2000 (Sigma), anti-TTRAP 1∶1000, anti-TRAF6 (Santa Cruz), anti-beta-actin 1∶5000 (Sigma). For detection, anti-mouse-HRP or anti-rabbit-HRP (Dako) in combination with ECL (GE Healthcare) were used.

### Statistical analysis

All experiments were repeated in triplicate or more. For stably transfected cells, at least two independent clones were used for each cell line in all experiments. Data represent the mean with standard deviation. When necessary, each group was compared individually with reference control group using Student's *t*-Test (Microsoft Excel software).

## Results

### TTRAP is present in cytoplasmic LBs and in the nucleolus of surviving dopaminergic neurons of PD post-mortem brains

**Figure 1:**
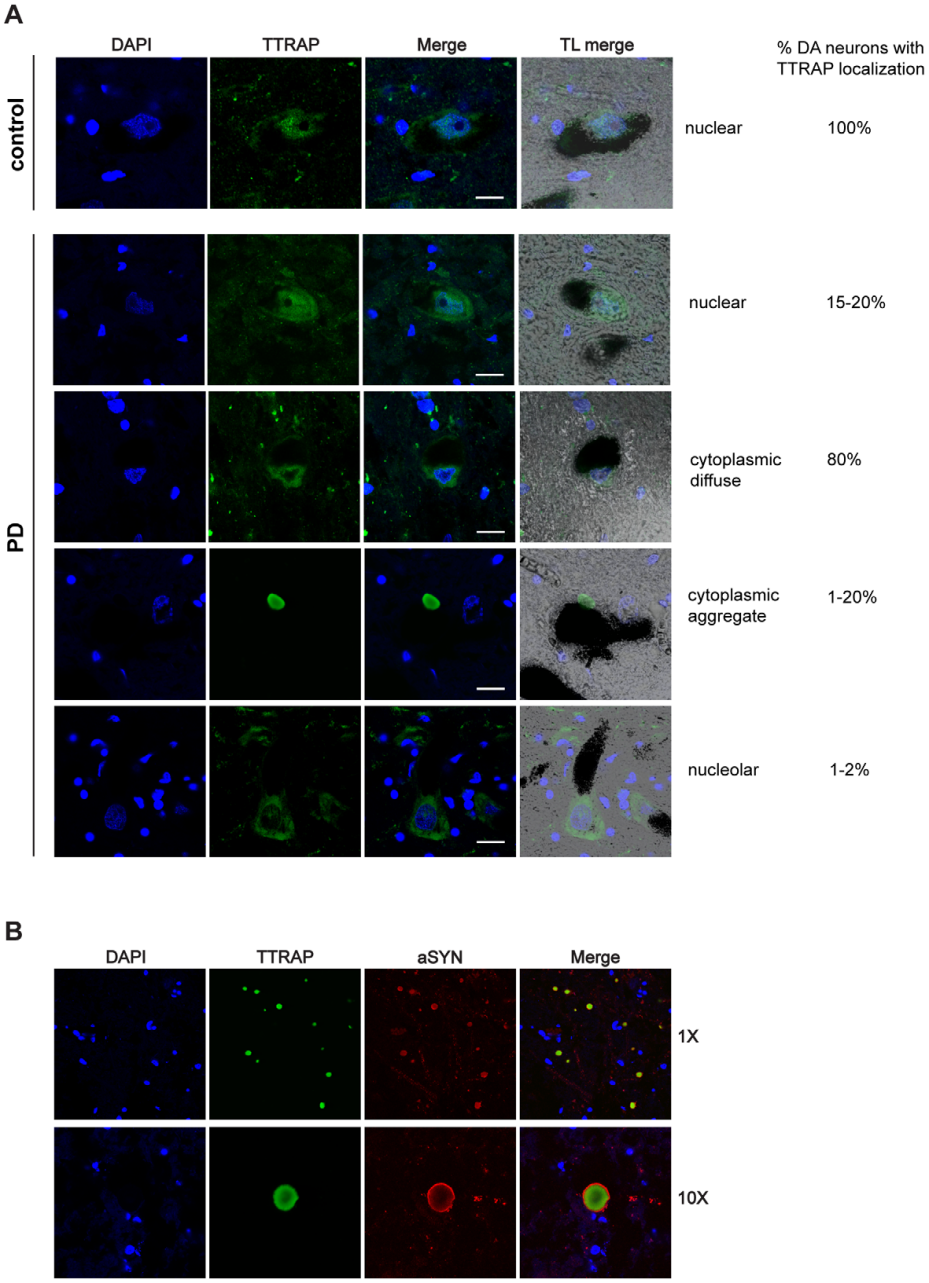
TTRAP is present in cytoplasmic Lewy bodies and in the nucleolus in surviving dopaminergic neurons in PD *post-mortem* brains. (A) TTRAP localization in normal and PD dopaminergic neurons. Cryo-sections of post-mortem brain tissues were taken from Substantia Nigra of healthy individuals and PD patients, as indicated. Immunohistochemistry was performed with anti-TTRAP antibody (green). Nuclei were stained with DAPI (blue). Dopaminergic neurons were identified by neuromelanin (black) in transmitted light (TL). Overlays of fluorescence (merge) and fluorescence with TL (TL merge) are shown. Panels are representative images showing TTRAP nuclear, cytoplasmic diffused, cytoplasmic aggregated and nucleolar localization. The percentage of dopaminergic neurons with nuclear, cytoplasmic or nucleolar TTRAP staining is indicated on the right. Bars, 15 µM. (B) TTRAP-containing cytoplasmic aggregates are Lewy bodies. Immunohistochemistry of Substantia Nigra from PD brains was performed with anti-TTRAP (green) and anti-alpha-synuclein (red) antibodies. Images at low (1×) and high (10×) magnification are shown.

To study TTRAP localization in sporadic PD, we performed immunohistochemical analysis in human post-mortem brains. A total of six PD patients and three controls were examined (Figure 1A). In normal conditions, TTRAP is expressed mainly in the nucleus of DA neurons, identified for the presence of neuromelanin. A weaker cytoplasmic staining is observed. Low levels of TTRAP expression can also be found in non-DA neurons. No staining was detected when control rabbit immunoglobulins were used (data not shown). In human brain sections of sporadic PD cases TTRAP expression was heterogeneous throughout mesencephalic cells identifying at least four phenotypes. While a small quantity of cells (15–20%) presented a nuclear distribution as in controls, in the majority of DA neurons (80%) TTRAP was exclusively relocalized to the cytoplasm, almost completely emptying the nucleus. In about 1–20% of the cells, TTRAP accumulated in large cytoplasmic aggregates. Double immunohistochemical analysis using anti-alpha-synuclein antibody demonstrated that these are *bona fide* LBs (Figure 1B). The patient-to-patient variability, in conjunction with an uneven distribution of aggregates in the Substantia Nigra, reflected the heterogeneous distribution of LBs in post-mortem brains. In addition, in 1–2% of surviving DA neurons, TTRAP diffuse cytoplasmic localization was concomitant with the presence of TTRAP in the nucleolus, as shown by lack of DAPI staining.

Altogether these data show that TTRAP is present in cytoplasmic LBs as well as in the nucleolus in surviving dopaminergic neurons of sporadic PD brains.

### L166P mutant DJ-1 inhibits TTRAP localization in nucleolar cavities

**Figure 2:**
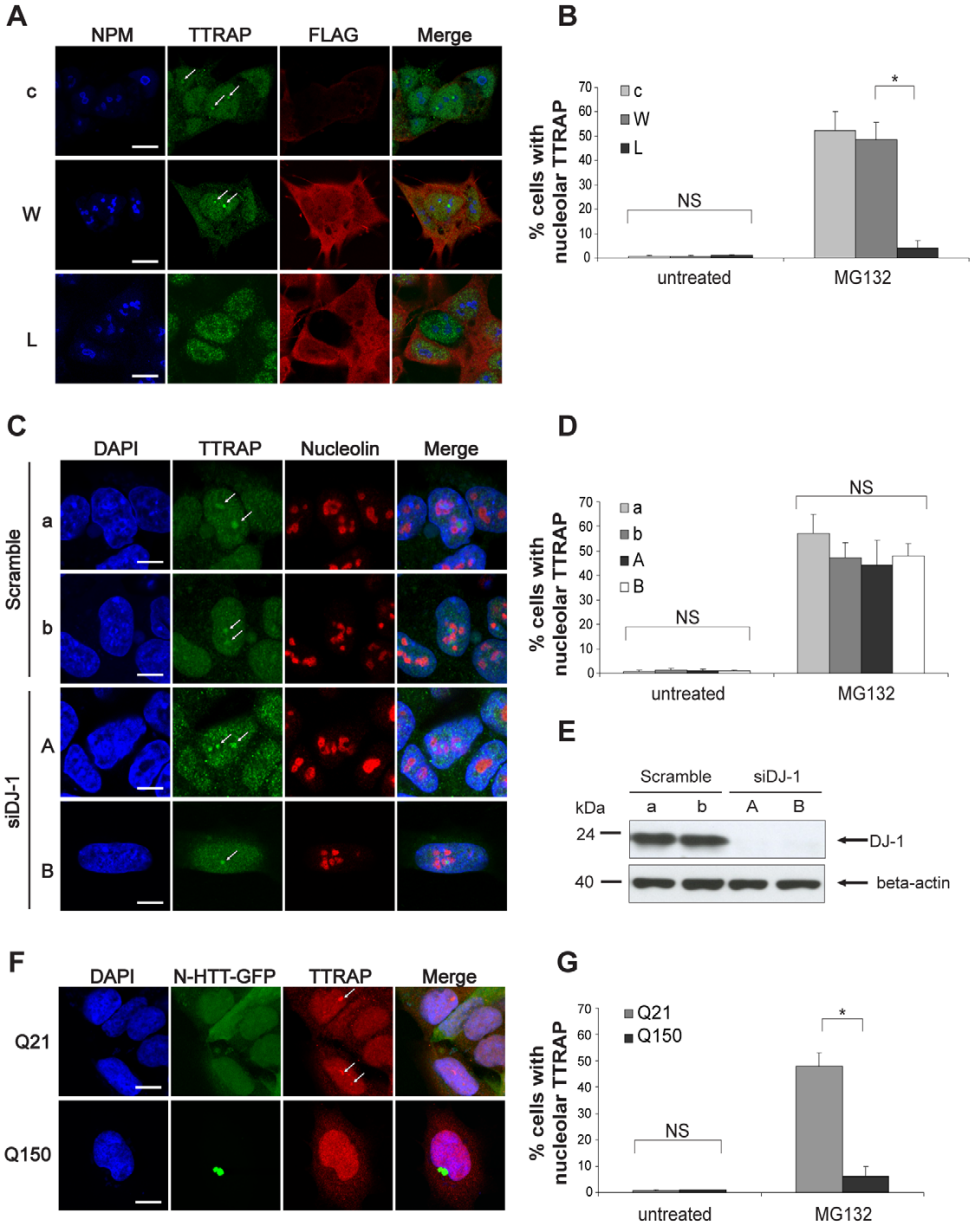
Mutant DJ-1 L166P inhibits TTRAP nucleolar localization after proteasome inhibition. (A) TTRAP nucleolar localization is inhibited in L166P expressing cells. SH-SY5Y cells stably transfected with empty vector (c), FLAG-DJ-1 wt (W) or L166P (L) were treated with 5 µM MG132 for 16 h. Triple immunofluorescence was performed with anti-NPM (blue), anti-TTRAP (green) and anti-FLAG (red) antibodies. Images are representatives of three independent experiments. Data have been confirmed on two independent clones for each cell line. Bars, 10 µm. (B) Quantification of TTRAP nucleolar localization. Cells as in A. At least 200 cells from two independent experiments were counted and scored for TTRAP in the nucleolus (*, p<0.05). (C) Localization of TTRAP is unaffected in cells depleted of endogenous DJ-1. SH-SY5Y cells stably expressing an inducible short-hairpin RNA targeting DJ-1 (siDJ-1, clones A and B) or a scramble shRNA control (scramble, clones a and b) were treated with doxycycline for 10 days to induce silencing of DJ-1 expression. Then cells were treated with 5 µM MG132 for 16 h. Immunofluorescence was performed with anti-TTRAP (green) and anti-NCL (red) antibodies. Nuclei were visualized with DAPI (blue). Images are representatives of two independent experiments. Bars, 10 µm. (D) Quantification of TTRAP nucleolar localization in siDJ-1 cells. Data were collected as in B. (NS, not-significant). (E) DJ-1 is efficiently depleted in siDJ-1 cells. Total protein lysates were prepared from cells as in C. Levels of endogenous DJ-1 were measured by western blot with anti-DJ-1 antibody. Beta-actin was detected as loading control. (F) TTRAP nucleolar localization is altered by mutant huntingtin. SH-SY5Y cells were transfected with huntingtin N-terminal fragment fused to GFP with WT (Gln^21^, Q21) or mutated (Gln^150^, Q150) polyglutamine expansion. Endogenous TTRAP was visualized by indirect immunofluorescence (red). N-HTT was visible by GFP autofluorescence. Nuclei were visualized with DAPI (blue). Bars, 10 µm. (F) Quantification of TTRAP nucleolar localization in N-HTT expressing cells. Data were collected as in B on GFP-positive cells (*, p<0.05; NS, not-significant).

When SH-SY5Y neuroblastoma cells are treated with proteasome inhibitors *in vitro*, TTRAP forms aggresome-like structures in the cytoplasm and in the nucleolus where it participates in rRNA biogenesis [20], [23]. In these conditions it binds L166P, the protein product of the most studied missense DJ-1 mutation in familial PD. To test whether L166P binding might have an effect on TTRAP localization, SH-SY5Y cells stably expressing FLAG-tagged wt DJ-1 or PD-associated L166P were treated with MG132. When nucleoli were visualized with the marker Nucleophosmin (NPM), TTRAP was detected in the nucleolus of about 60% of control cells upon proteasome block (Figure 2A, 2B) [20]. This localization was also observed when more specific inhibitors of proteasome activity were used like epoxomicin and lactacystin (Figure S1). No staining was observed in cells with knock-down TTRAP expression (Figure S1). While overexpression of wt DJ-1 had no effect on TTRAP nucleolar localization, the presence of misfolded L166P mutant almost completely inhibited TTRAP recruitment to the nucleolus (Figure 2A, 2B and figure S2). No differences in TTRAP localization were observed in untreated conditions, thus indicating that overexpression of wt or mutant DJ-1 per se does not alter TTRAP subcellular distribution (Figure 2B and Figure S2). Since L166P DJ-1 is expressed at lower levels than wt protein and is generally associated to a loss-of-function phenotype, we asked whether silencing DJ-1 expression in SH-SY5Y cells could recapitulate the phenotype observed in L166P expressing cells. We performed similar immuno-fluorescence experiments in SH-SY5Y cells with inducible loss of DJ-1 expression [11] by using two independent siDJ-1 (A, B) and scramble (a, b) clones. The lack of DJ-1 protein did not affect TTRAP localization, neither in untreated or MG132-treated cells (Figure 2C, 2D, 2E and Figure S3).

To evaluate whether TTRAP exclusion from the nucleolus was a phenotype common to other misfolded proteins involved in neurodegenerative diseases, we studied TTRAP localization in a cellular model of Huntington's disease. SH-SY5Y cells were transfected with huntingtin amino-terminal fragment (residues 1–171) fused to green fluorescent protein (N-HTT-GFP) with either a physiological (Gln^21^) or a pathological (Gln^150^) polyglutamine (polyQ) stretch. N-HTT aggregates triggered by polyQ expansion inhibited TTRAP nucleolar localization upon proteasome impairment (Figure 2F, 2G), while no effects were observed when wt Gln^21^ N-HTT was used.

These data indicate that misfolding-causing mutations in neurodegenerative diseases impact TTRAP nucleolar localization upon proteasome inhibition.

### L166P alters rRNA biogenesis in response to proteasome inhibition

**Figure 3:**
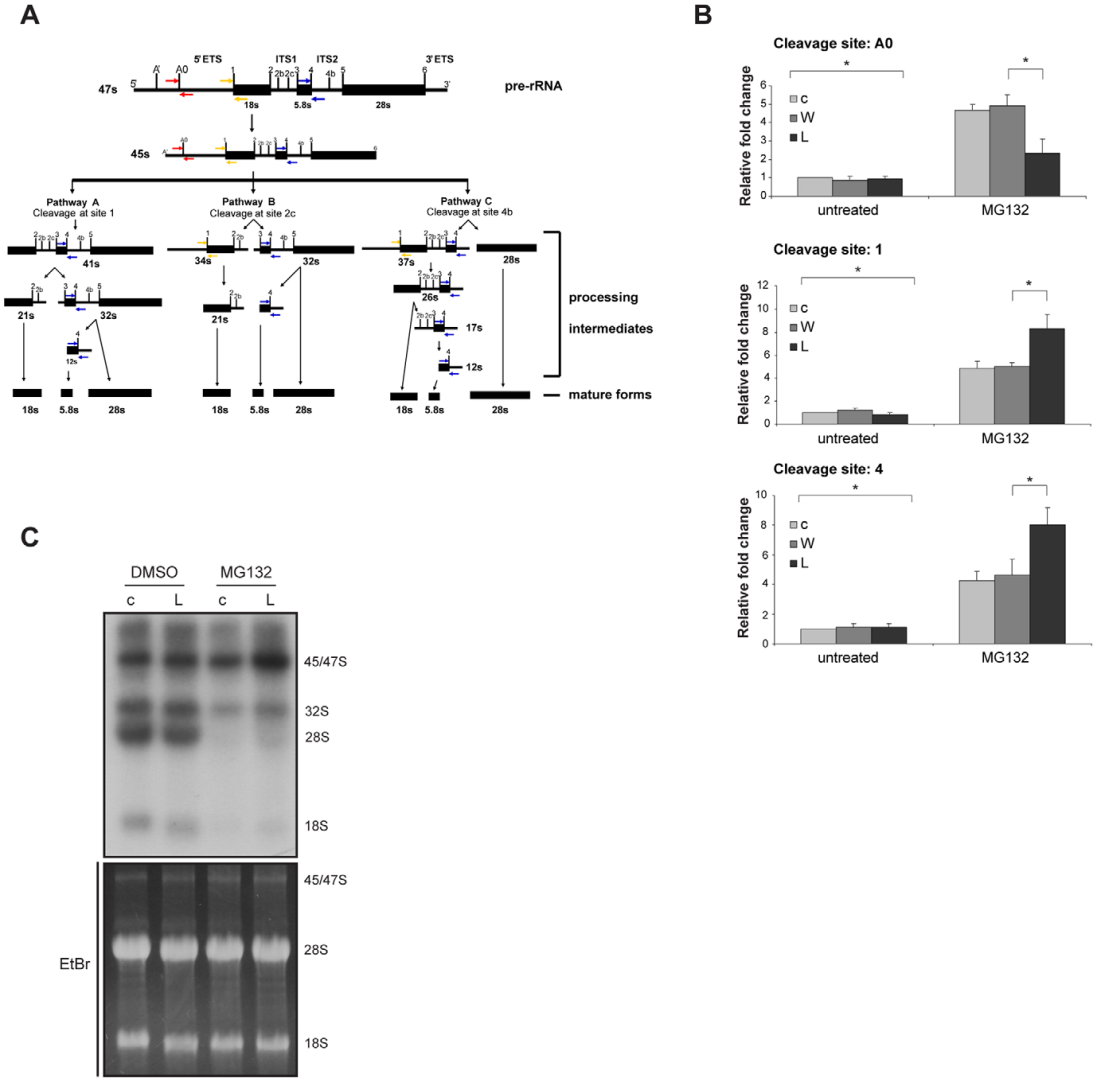
Processing of rRNA is altered in L166P overexpressing cells. (A) Schematic diagram showing pre-rRNA structure and processing steps. Positioning of A0, 1 and 4 cleavage sites on rRNA processing intermediates is shown. ETS, external transcribed spacer; ITS, internal transcribed spacer. (B) Analysis of steady-state levels of rRNA precursor and processing intermediates. SH-SY5Y cells stably expressing wt DJ-1 (W), L166P (L) and empty vector (c) were treated with Bars, 5 µM MG132 for 16 h, or left untreated. Total RNA was extracted and levels of pre-rRNA and processing intermediates were analyzed by qPCR with primers targeting A0 , 1 and 4 cleavage sites. Standard deviations are calculated on four replicas from two independent experiments. *, p<0.05. (C) Analysis of rRNA processing by metabolic labeling. L166P (L) and control (c) cells were treated as in A. After treatment, cells were pulse-labeled with ^32^P-orthophosphate for 1 h and chased with cold medium for 3 h. RNA was extracted and equal quantities were separated on denaturing agarose gel. Nascent rRNA was visualized by autoradiography after gel drying (upper panel). rRNA processing intermediates and mature forms are indicated on the right. Loading was verified with ethidium bromide staining (lower panel).

Since we have previously found that nucleolar TTRAP regulates rRNA biogenesis in conditions of proteasome inhibition [20], we analyzed the levels of precursor rRNA molecules (pre-rRNA) and of rRNA processing intermediates in SH-SY5Y stable cells for wt DJ-1, mutant L166P and empty vector as control. Cells were treated with MG132 and rRNA biogenesis was measured by qPCR with A0, 1 and 4 primers as previously described [28], [29], [30] [20]. A scheme of rRNA processing events and positioning of qPCR primers on processing intermediates is shown in figure 3A. In untreated conditions, A0, 1 and 4 probes detected no changes dependent on wt or mutant DJ-1 overexpression (Figure 3B). Upon proteasome inhibition, the levels of pre-rRNA containing A0 sites were significantly increased (p<0.05). This pattern was maintained in cells expressing wt DJ-1. On the contrary, concomitant with the disappearance of nucleolar TTRAP, in L166P cells the quantity of A0 sites was strongly reduced (p<0.05). Furthermore, the amount of rRNA processing intermediates, measured by oligonucleotides targeting 1 and 4 cleavage sites, dramatically increased in L166P cells compared to wt DJ-1 and controls (Figure 3B). No detectable alterations in pre-rRNA levels were observed in inducible si-DJ-1 cells (Figure S4). Importantly, no gross alterations in the structure of nucleoli were induced by overexpression of L166P as proved with NPM staining (Figure S5).

To further investigate ribosome biogenesis in mutant DJ-1 cells, we measured nascent rRNA by pulse-chase labeling with ^32^P-orthophosphate. Untreated cells were used as controls. Metabolically labeled rRNA species were visualized by autoradiography (Figure 3C, upper panel) and loading was verified with ethidium bromide (Figure 3C, lower panel). No differences in rRNA processing were detected in untreated conditions between control and mutant DJ-1 cells, thus confirming qPCR data. While proteasome inhibition caused a marked reduction of 28S and 18S mature forms in both cell lines, mutant DJ-1 L166P expression caused an increase in pre-rRNA precursors (47S/45S) and an accumulation of the 32S processing intermediate.

Altogether, these results suggest that misfolded DJ-1 expression has an impact both on the initial events of transcription and on the conversion of intermediate to mature forms (47S to 45S and 32S to 28S). This phenotype resembles the one observed in neuroblastoma cells lacking TTRAP expression [20]. Analysis of cell proliferation in untreated and treated conditions could not detect any difference between control and mutant cells (Figure S6). A small, but significant, decrease in cell viability could be observed in L166P cells exposed to MG132 as compared to control cells (Figure S6).

### L166P-mediated exclusion of TTRAP from nucleolar cavities does not depend on PML

**Figure 4:**
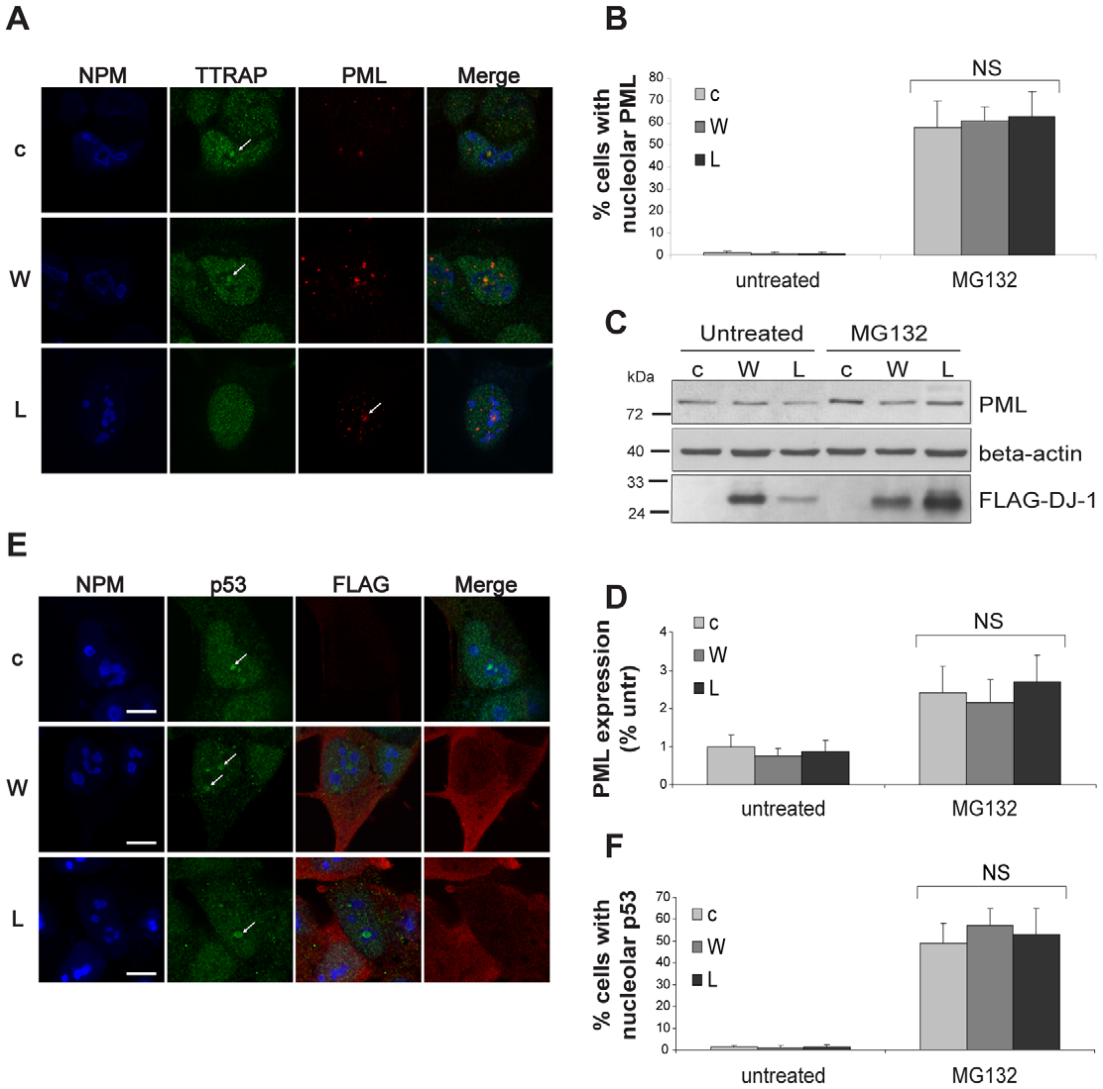
Mutant DJ-1 L166P does not inhibit nucleolar localization of PML and p53 upon proteotoxic stress. (A) Nucleolar localization of PML. SH-SY5Y cells stably expressing wt DJ-1 (W), L166P (L) or empty vector (c) were treated with 5 µM MG132 for 16 h. Endogenous TTRAP (green), PML (red) and NPM (blue) localization was analyzed by triple immunofluorescence. Images are representatives of two separate experiments performed on two independent clones for each cell line. (B) Quantification of PML nucleolar localization. Cells were treated as in A. 200 cells from two independent experiments were counted and scored for PML in the nucleolus (*, p<0.05). (C) Mutant DJ-1 L166P does not affect levels of endogenous PML. SH-SY5Y cells stably expressing wt DJ-1 (W), L166P (L) or empty vector (c) were treated with 5 µM MG132 for 16 h or left untreated, as indicated. The expression of endogenous PML and overexpressed FLAG-DJ-1 were measured with anti-PML and anti-FLAG antibodies, respectively. Beta-actin was detected as loading control. (D) Quantification of PML levels. Densitometric analysis of protein bands was performed on two independent experiments. Relative expression of endogenous PML was normalized to beta-actin. Data are expressed as the percentage of untreated condition in empty cells (c). (E) Nucleolar localization of p53. Cells were exactly as in A. Localization of endogenous p53 (green), NPM (blue) and FLAG-DJ-1 (red) were analyzed by triple immunofluorescence. Bars, 10 µm. (F) Quantification of p53 nucleolar localization. Analysis was performed as in B (NS, non significant).

The presence of PML and the integrity of PML-NBs in nucleolar cavities are fundamental for TTRAP to be recruited to the nucleolus in response to proteasome inhibition [20]. Therefore, we asked whether L166P-mediated exclusion of TTRAP from nucleolar cavities might depend on L166P action on PML-NBs. SH-SY5Y cells expressing wt, L166P or an empty vector were treated with MG132 and PML localization was followed by immunofluorescence using NPM as nucleolar marker (Figure 4A). The number of cells with nucleolar PML-NBs was scored in duplicates in two independent clones for each cell line (at least 100 cells per experiment) (Figure 4B). Total levels of PML protein were measured by western blot analysis (Figure 4C, 4D). We found that L166P, similarly to wt and control cells, did not impact PML expression or PML-NBs accumulation in nucleolar cavities. Therefore, L166P inhibition of TTRAP nucleolar accumulation is independent from PML. To further support this model, we also checked whether MG132-induced nucleolar localization of p53, another marker of nucleolar cavities, was altered by L166P. Similarly to PML staining, we couldn't observe any change in p53 localization induced by overexpression of misfolded mutant DJ-1, or in controls (Figure 4E, 4F). No changes in TTRAP, PML and p53 localization were observed in untreated cells (Figure S7).

Altogether our data indicate that L166P overexpression does not impact the overall structure of nucleolar cavities but specifically alters nucleolar localization of L166P-binding protein TTRAP.

### L166P enhances TTRAP accumulation into insoluble cytoplasmic aggresomes

**Figure 5:**
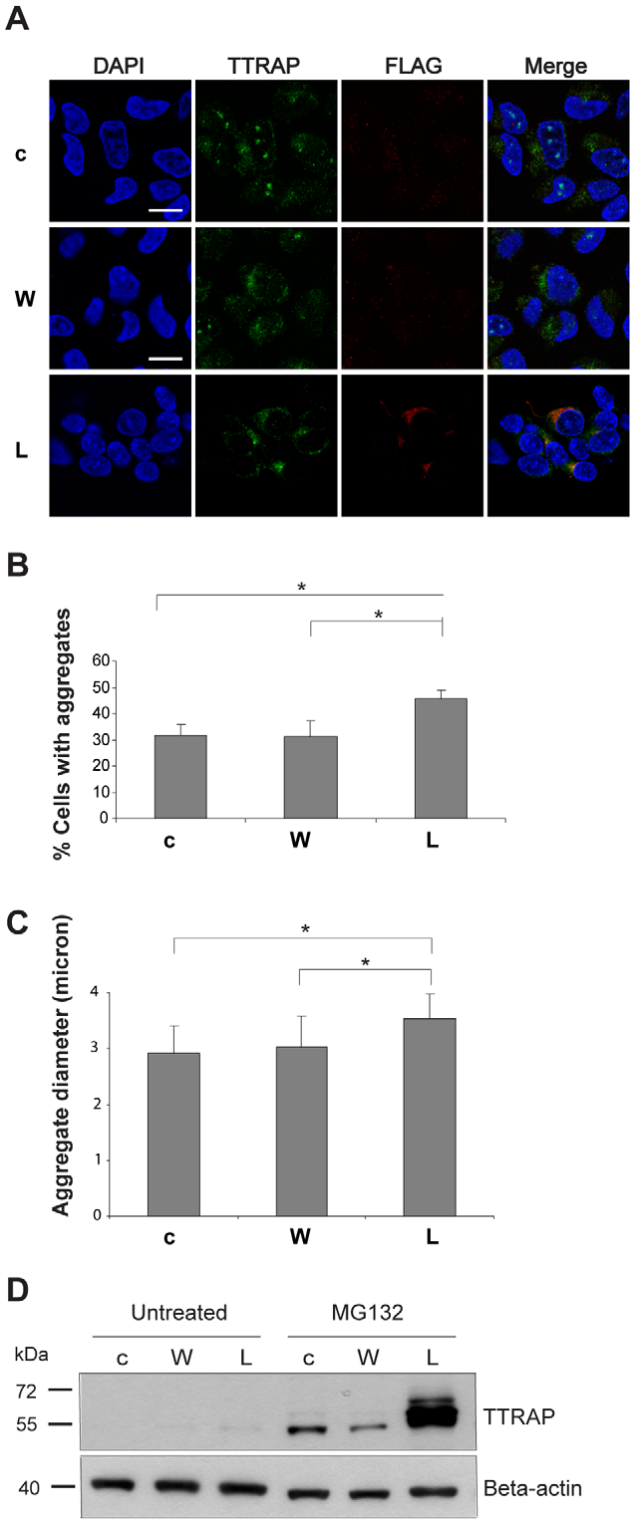
L166P enhances TTRAP accumulation into insoluble cytoplasmic aggresomes. (A) Cytoplasmic localization of TTRAP. SH-SY5Y cells stably expressing wt DJ-1 (W), L166P (L) or empty vector (c) were treated with 5 µM MG132 for 16 h. Before fixation, cells were permeabilized with Triton X-100 and double immunofluorescence was performed with TTRAP (green) and anti-FLAG (red) antibodies. Nuclei were visualized with DAPI. Confocal images from two independent experiments were scored for percentage of cells with aggregates (B), and aggregate average size (C). At least 50 cells per experimental condition were counted (*, p<0.05). (D) Mutant DJ-1 L166P promotes accumulation of TTRAP in insoluble fraction. Cells were as in A. After treatment with MG132, cells were lysed, and Triton X-100 insoluble fraction was prepared. Endogenous TTRAP was analyzed by western blotting with anti-TTRAP antibody. Protein loading was controlled by beta-actin.

As MG132 treatment accumulates TTRAP into cytoplasmic insoluble aggresome-like structures [23], we analyzed TTRAP localization in cells that express wt DJ-1 or L166P after permeabilization with Triton X-100 before fixation. In all cell lines analyzed, endogenous TTRAP formed aggresome-like structures in the cytoplasm, as expected (Figure 5A), whereas no aggresomes were visible in untreated conditions (data not shown). Interestingly, overexpression of L166P increased the percentage of cells with TTRAP-containing cytoplasmic aggregates and promoted the formation of inclusions with an average larger size (Figure 5A, 5B, 5C).

In about half of SH-SY5Y cells stably transfected with DJ-1 wt or empty vector, TTRAP was also present in the insoluble fraction of the nucleolus. As expected, TTRAP accumulation in insoluble nucleolar granules was almost completely inhibited in presence of L166P mutant (Figure 5A). Interestingly, L166P itself was present in TTRAP-positive cytoplasmic aggregates (figure 5A), whereas no DJ-1 aggregates were visible in wt overexpressing cells.

Since mutant L166P promotes TTRAP accumulation in cytoplasmic aggregates upon MG132 treatment, we analyzed TTRAP distribution into insoluble fractions by western blot analysis, proving a more evident accumulation in L166P-expressing cells (Figure 5D) [23].

Altogether, these data demonstrate that L166P depletes TTRAP from nucleolar cavities and enhances its recruitment into L166P-containing insoluble cytoplasmic aggregates.

### TRAF6 E3 ligase activity contributes to L166P-mediated exclusion of TTRAP from nucleolar cavities

**Figure 6:**
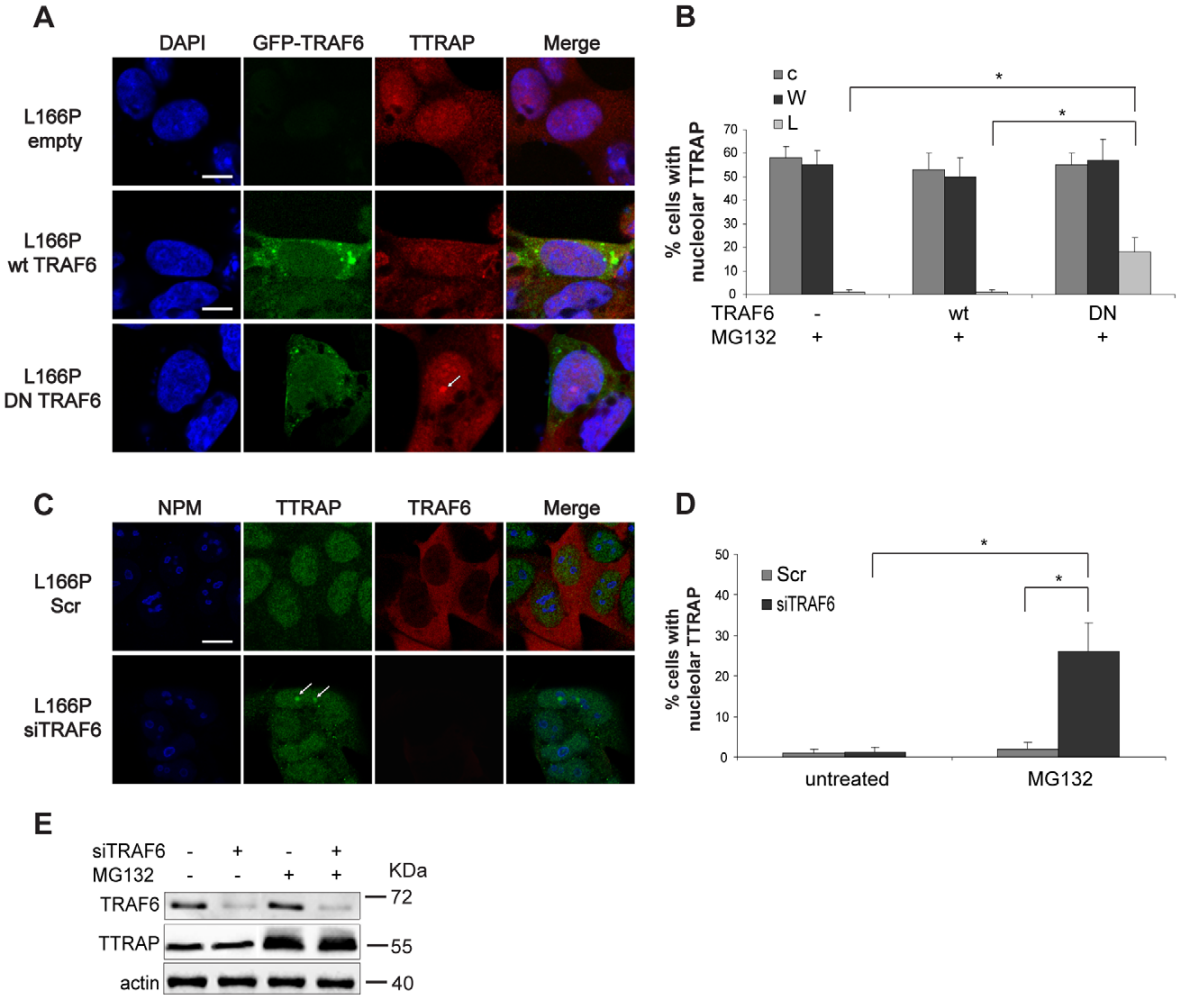
TRAF6 E3 ligase activity contributes to L166P-mediated exclusion of TTRAP from nucleolar cavities. (A) Expression of TRAF6 DN partially rescues TTRAP nucleolar localization in L166P cells. SH-SY5Y cells stably expressing mutant DJ-1 were transfected with GFP-tagged TRAF6 (wt or deleted of the N-terminus, DN), or with empty vector control, as indicated. After 24 h from transfection, cells were treated for 16 h with MG132. TTRAP localization was followed by indirect immunofluorescence coupled with GFP autofluorescence. Bars, 10 µm. (B) Quantification of TTRAP nucleolar localization. Cells were as in A. At least 100 transfected cells with comparable GFP levels from two independent experiments were counted and scored for TTRAP in the nucleolus (*, p<0.05). (C) Knock-down endogenous TRAF6 expression partially rescues TTRAP nucleolar localization. SH-SY5Y cells stably transfected with mutant DJ-1 L166Pwere transfected with siRNA oligonucleotides targeting endogenous TRAF6 (siTRAF6) or with a scramble control siRNA (Scr). After 72 h from transfection, cells were treated for 16 h with MG132. Immunofluorescence was performed with anti-TTRAP (green), anti-TRAF6 (red), anti-NPM (blue). Bars, 15 µm. (D) Quantification of TTRAP nucleolar localization. Cells were as in C. Analysis was performed as in B on cells with reduced TRAF6 expression (*, p<0.05). (E) Total cell lysates were prepared from SH-SY5Y cells transfected as in C. Cells were treated with MG132 for 16 h or left untreated. Expression of endogenous TRAF6 and TTRAP was measured with specific antibodies. Protein loading was controlled by beta-actin. Molecular weight markers (MWM) are indicated for each gel (kDa). Images are representative of two independent experiments.

Since TTRAP and L166P co-localize in cytoplasmic insoluble inclusions, we hypothesized that factors that promote L166P aggregation might control TTRAP distribution between the cytoplasm and the nucleolus. In a previous work, we have shown that atypical ubiquitination by the E3 ligase TRAF6 enhances L166P aggregate formation [15]. Therefore, we tested whether TRAF6 E3 ligase activity might contribute to L166P-induced TTRAP mislocalization. To this purpose we took advantage of a TRAF6 mutant deleted of the N-terminal E3 ligase RING domain (TRAF6 DN), that acts as dominant negative [31]. We transfected SH-SY5Y cells stably expressing L166P with TRAF6 DN or wt fused to GFP (Figure 6A, 6B). Cells expressing wt DJ-1 or empty vector were used as controls. TTRAP localization in nucleolar cavities was scored only in those cells with equivalent expression of transfected constructs. Overexpression of wt TRAF6 had no effect on TTRAP exclusion from nucleolar cavities induced by L166P. Instead, TTRAP nucleolar localization was restored in about 20% of cells when TRAF6 DN was used (Figure 6A, 6B). This phenotype was only partially penetrant. Overexpression of TRAF6 or TRAF6 DN in stable cells for wt DJ-1 or empty vector had no effects on TTRAP localization (Figure 6B). No differences in TTRAP localization were observed in untreated condition (Figure S8), thus indicating that overexpression of wt or mutant TRAF6 per se does not alter TTRAP subcellular distribution.

To further support the role of TRAF6 in excluding TTRAP from nucleolar cavities, endogenous TRAF6 was silenced in L166P-expressing cells with specific siRNA oligonucleotides. Scramble siRNA and wt DJ-1 cells were used as controls. Knocking-down TRAF6 expression recapitulated the phenotype observed with TRAF6-DN, with partial recovery of nucleolar TTRAP localization (Figure 6C, 6D and 6E). In line with partial rescue of TTRAP nucleolar localization, knockdown of TRAF6 could also partially recover rRNA biogenesis defects in mutant DJ-1 cells exposed to proteasome inhibition, although not all processing intermediates were affected to the same extent (Figure S9); this observation is also compatible with a DJ-1/TTRAP independent role of TRAF6 in regulating ribosome biogenesis in response to proteotoxic stress.

In any case, our data indicate that TRAF6 E3 ligase activity contributes to TTRAP partitioning between cytoplasmic and nucleolar aggregates in the presence of misfolded mutant DJ-1.

## Discussion

L166P presents a structural rearrangement that interferes with dimer formation favoring oligomerization and protein instability [13], [32]. This led to the hypothesis that DJ-1 deletions and missense mutations share a common phenotype based on a loss-of-function model. However, increasing evidences indicate that missense mutations like L166P may also present gain-of-function properties. L166P has exerted dominant-negative effects on the antioxidant wt DJ-1 activity [33]. Gene profiling experiments in DJ-1 KO and in L166P-NIH3T3 cells [34] have shown that L166P affects the expression of a larger number of genes with no common genes between these two conditions. Furthermore, tau promoter was directly upregulated by L166P, in conditions when wt DJ-1 was acting as a repressor. In this context, we have previously shown that upon proteasome impairment L166P blocked the neuroprotective property of TTRAP to MG132 treatment inducing JNK and p38 MAPKs apoptotic pathways [23].

Here we provide evidences for a novel pathogenic mechanism of mutant DJ-1 mediated by the sequestration of TTRAP. Upon proteasome inhibition, L166P triggers alterations of rRNA biogenesis by selectively excluding TTRAP from nucleolar cavities and sequestering it into cytoplasmic aggregates. Inhibition of TTRAP nucleolar localization by accumulated misfolded proteins is not restricted to mutant DJ-1, but can also be observed for aggregates of mutant huntingtin.

rRNA biogenesis is a complex process that requires the transcription of rDNA genes by polymerase I, nucleolytic cleavages and chemical modification of precursor rRNAs and ultimately leads to the assembly of mature ribosomes [35]. It is subjected to rigorous quality control mechanisms [36], [37] and stressful events elicit homeostatic responses that involve the majority of its steps [38], [39]. We have previously found that TTRAP regulates rRNA biogenesis under proteasome impairment. TTRAP down-regulation leads to a decrease of pre-rRNA and a concomitant increase of processing species thus suggesting it might function at multiple steps [20]. Accumulation of processing intermediates might be due to impaired cleavage or to reduced degradation of cleaved fragments, while the effects on pre-rRNA levels may unveil a role of TTRAP at rDNA loci or may be secondary to changes in rRNA processing [40].

Here we show that misfolded mutant DJ-1 expression recapitulates this phenotype affecting the initial events of transcription and the conversion of intermediate to mature forms (47S to 45S and 32S to 28S). While both qPCR and pulse-chase labeling with ^32^P-orthophosphate indicate an increase in the amount of rRNA processing intermediates, the quantity of A0 sites was strongly reduced when measured with qPCR while seemed increased in pulse-chase experiment. To reconcile these data we hypothesize that PCR amplification of the 5′ ETS region of rRNA precursors may detect illegitimate small RNA species due to abortive transcription initiation that are not visible by gel electrophoresis.

Several human genetic diseases, collectively defined as ribosomopathies, are caused by mutations in genes involved rRNA biogenesis and ribosome assembly [41]. Surprisingly, few studies so far have analyzed the status of protein synthesis, ribosome biogenesis and of the nucleolus in human post-mortem brains of neurodegenerative diseases. Alteration of protein synthesis capability due to ribosome dysfunction has been shown to be an early event in Alzheimer's disease [42]. A nucleolar protein involved in ribosome biogenesis was found induced in post-mortem brains of Huntington's disease patients [43]. Nucleolin was identified as an interactor of the PD-associated proteins DJ-1 and alpha-synuclein and its protein expression was found reduced in Substantia Nigra of human PD brains [25]. More recently, genetic ablation of rRNA transcription caused an alteration of the nucleolus architecture and ultimately neurodegeneration both in hippocampal and dopaminergic neurons [26], [44].

An analysis of rRNA biogenesis in post-mortem brains of familial PD cases and in animal models of the disease is thus needed. Interestingly, the association of TTRAP to the nucleolus of DA cells in sporadic PD brains increases the list of changes in the nucleolar composition in PD. Their functional effects remain to be investigated.

Recently, nucleolar aggregates have been identified as a novel form of nuclear stress bodies that occupy nucleolar cavities [20], [28]. They contain polyadenylated RNA, unconjugated ubiquitin and several nucleoplasmic proteasome target proteins [28]. Their formation was relieved by an excess of free cytoplasmic ubiquitin and occurred with both wt and triple mutant ubiquitins lacking K48, K29 and K63 lysines, proving that atypical ubiquitination is involved. This led to the hypothesis that a cross-talk between cytoplasmic and nucleolar aggregates regulates the subcellular localization of atypically ubiquitinated misfolded proteins.

The partitioning of TTRAP between nucleolar and cytoplasmic aggregates is modulated by TRAF6. This E3 ligase binds both TTRAP and mutant DJ-1 promoting atypical ubiquitination of L166P and its accumulation in cytoplasmic aggregates [15]. When TRAF6 ligase activity is lacking, L166P inclusions are smaller and TTRAP is relieved to relocate, at least in part, into the nucleolus. Other E3 ubiquitin ligases, such as parkin [45], have been demonstrated to act on mutant DJ-1, possibly accounting for additional TRAF6-independent regulation. TRAF6, as other E3 ubiquitin ligases, is accumulated in LBs [15]. In this context, the aberrant localization of TTRAP in LBs and in the nucleolus of sporadic PD post-mortem brains may suggest the existence of this regulatory pathway *in vivo*. On the other hand, the impact of TTRAP sequestration and consequent alteration in rRNA biogenesis in familial cases will ultimately depend on the level of L166P protein, which remains unknown. In summary this work demonstrates a novel role for misfolded mutant DJ-1 in the homeostasis of rRNA biogenesis and suggests the existence of a functional interplay between cytoplasmic and nucleolar aggregates that may regulate nucleolar functions in neurodegenerative diseases.

## Supporting Information

Figure S1 **Quantitative analysis of TTRAP nucleolar localization.** (A) TTRAP localizes to the nucleolus upon proteotoxic stress. SH-SY5Y cells were treated with increasing concentration of Epoxomycin or Lactacystin, as indicated. Untreated cells were used as controls. TTRAP was visualized with indirect immunofluorescence with anti-TTRAP antibody (green). Nuclei were visualized with DAPI (blue). Nucleolar TTRAP was scored in DAPI-negative regions in >100 cells. Representative images are shown for cells treated with 1 µM Epoxomycin and 25 µM Lactacystin. (*, p<0.05). (B) TTRAP staining is specific. SH-SY5Y cells stably expressing a short-hairpin RNA targeting TTRAP (siTTRAP #1 and #2) or a scrambled shRNA control (scramble #1 and #2) were treated for 16 h with 5 µM MG132. TTRAP (green) and nuclei (blue) were stained as in A.
(TIF)

Figure S2 **Altered TTRAP nucleolar localization in L166P mutant cells upon treatment with MG132.** SH-SY5Y cells stably transfected with empty vector (c), FLAG-DJ-1 wt (W) or L166P (L) were treated with 5 µM MG132 for 16 h or left untreated. TTRAP localization was analyzed by immunofluorescence with anti-TTRAP (green) and anti-FLAG (red) antibodies. Nuclei were visualized by DAPI staining (blue). Low magnification images are shown. Nucleolar TTRAP is evident in DAPI-negative regions of the nucleus (white arrows). Images are representatives of three independent experiments from two independent clones for each cell line.
(TIF)

Figure S3 **Analysis of DJ-1 expression in siDJ-1 and scramble SH-SY5Y cells.** SH-SY5Y cells stably expressing a doxycyclin-inducible short-hairpin targeting DJ-1 (siDJ-1, clones A and B) or a scramble shRNA control (scramble, a and b) were treated with doxycycline for 10 days. Endogenous DJ-1 expression was analyzed by immunofluorescence with anti-DJ-1 antibody.
(TIF)

Figure S4 **Depletion of DJ-1 does not alter transcription and processing of ribosomal RNA.** SH-SY5Y stably expressing a doxycyclin-inducible shRNA targeting DJ-1 (siDJ-1, clones A and B) or a scramble shRNA control (scramble, a and b) were induced 10 days with doxyciclin and then treated with 5 µM MG132 for 16 h, or left untreated. Total RNA was extracted and levels of pre-rRNA and processing intermediates were analyzed by qPCR. Amplicons are those described in figure 3. Standard deviations are calculated from two independent experiments. Differences between a, b, A and B are not statistically significant (*, P<0.05. NS, not significant).
(TIF)

Figure S5 **Analysis of the effects of wild-type and mutant DJ-1 on nucleolar integrity.** SH-SY5Y cells stably expressing wt DJ-1 (W), L166P (L) or empty vector (c) were treated with 5 µM MG132 for 16 h. Immunofluorescence was performed with anti-NPM antibody and NPM nucleoplasmic staining was measured with ImageJ software on a randomly selected area. Background fluorescence was quantified from an area placed outside the cells and was subtracted for each signal. At least 100 cells from two separate experiments were counted (NS, not statistically significant). Representative zoomed images are shown for each cell line.
(TIF)

Figure S6 **Analysis of the effects of mutant DJ-1 L166P on cell proliferation and viability.** (A) Expression of L166P does not affect the cell cycle. SH-SY5Y cells stably expressing the L166P mutant and control cells were treated for 16 h with 5 µM MG132, or DMSO as control. Cell proliferation was analyzed by flow-cytometry (FACS) after propidium iodide (PI) staining. Graphs show the overlay of representative FACS profiles for each sample. (B) Quantification of the cell cycle distribution in control and L166P cells treated as in A. Data are from three independent experiments (error bars, standard deviation). (C) Expression of L166P DJ-1 moderately sensitizes cells to death induced by proteasome inhibition. Identical numbers of L166P and control cells were seeded in 96-well plates. After 24 hours, cells were treated with DMSO or 5 µM MG132 for additional 16 h. Cell viability was measured by WST-1 assay. Data are normalized to WST activity in untreated cells. Standard deviations are calculated from four independent experiments (*, p<0.05).
(TIF)

Figure S7 **Localization of PML and p53 is not affected by the expression of DJ-1 L166P mutant in untreated cells.** (A) Localization of PML. SH-SY5Y cells stably expressing FLAG-tagged DJ-1 wt (W), or mutant (L) or empty vector (c) were stained by triple immunofluorescence with anti-TTRAP (green), anti-PML (red) and anti-NPM (blue) antibodies. (B) Localization of p53. Cells were stained by triple immunofluorescence with anti-NPM (blue), anti-p53 (green) and anti-FLAG (red) antibodies. Bars, 10 µm.
(TIF)

Figure S8 **TRAF6 expression does not alter TTRAP localization in untreated cells.** SH-SY5Y stably expressing L166P were transfected with GFP-TRAF6 (wt and DN), as indicated. Cells were left untreated. TTRAP localization was analyzed by immunofluorescence with anti-TTRAP (red) antibody. Bars, 10 µm.
(TIF)

Figure S9 **Analysis of rRNA biogenesis in mutant DJ-1 L166P cells with knock-down of TRAF6 expression.** SH-SY5Y cells stably transfected with mutant DJ-1 L166P were transfected with oligonucleotides targeting endogenous TRAF6 (siRNA TRAF6) or a scramble control sequence (siRNA Scramble). After 72 h from transfection, cells were treated for 16 h with MG132 or DMSO as control. Total RNA was extracted and levels of pre-rRNA and processing intermediates were analyzed by qPCR with primers targeting A0, 1 and 4 cleavage sites, as indicated. Efficiency of TRAF6 knock-down was monitored with specific primers (TRAF6). Standard deviations are calculated from four independent experiments (*, P<0.05).
(TIF)
