# Supplementary figures and images for: Parkinson's Disease DJ-1 L166P Alters rRNA Biogenesis by Exclusion of TTRAP from the Nucleolus and Sequestration into Cytoplasmic Aggregates via TRAF6
Source: PLoS One. 2012 Apr 20;7(4):e35051. doi: 10.1371/journal.pone.0035051 (PMC3332112; doi:10.1371/journal.pone.0035051)

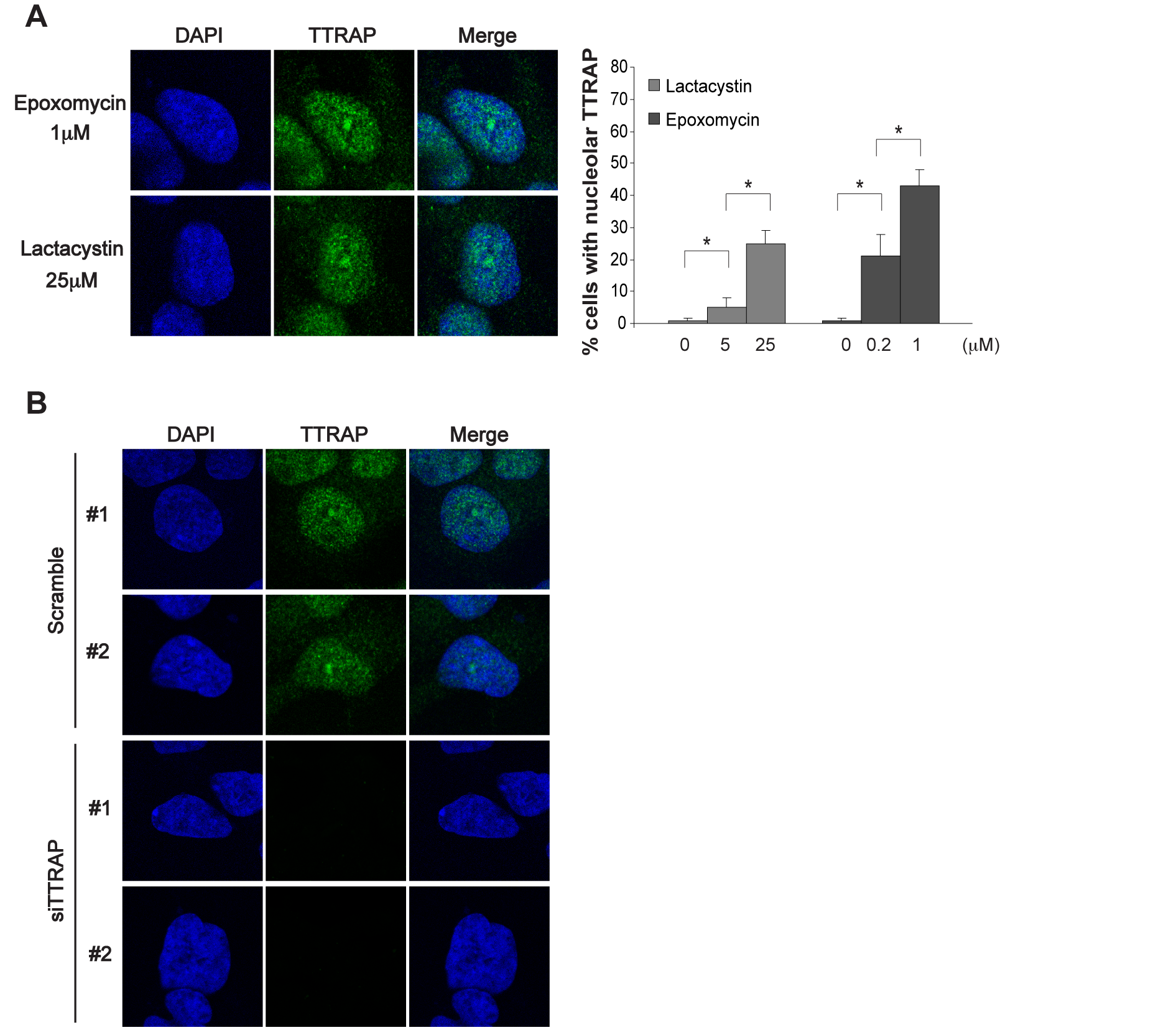

Supplement: Figure S1 — Quantitative analysis of TTRAP nucleolar localization. (A) TTRAP localizes to the nucleolus upon proteotoxic stress. SH-SY5Y cells were treated with increasing concentration of Epoxomycin or Lactacystin, as indicated. Untreated cells were used as controls. TTRAP was visualized with indirect immunofluorescence with anti-TTRAP antibody (green). Nuclei were visualized with DAPI (blue). Nucleolar TTRAP was scored in DAPI-negative regions in >100 cells. Representative images are shown for cells treated with 1 µM Epoxomycin and 25 µM Lactacystin. (*, p<0.05). (B) TTRAP staining is specific. SH-SY5Y cells stably expressing a short-hairpin RNA targeting TTRAP (siTTRAP #1 and #2) or a scrambled shRNA control (scramble #1 and #2) were treated for 16 h with 5 µM MG132. TTRAP (green) and nuclei (blue) were stained as in A. (TIF) [file pone.0035051.s001.tif]

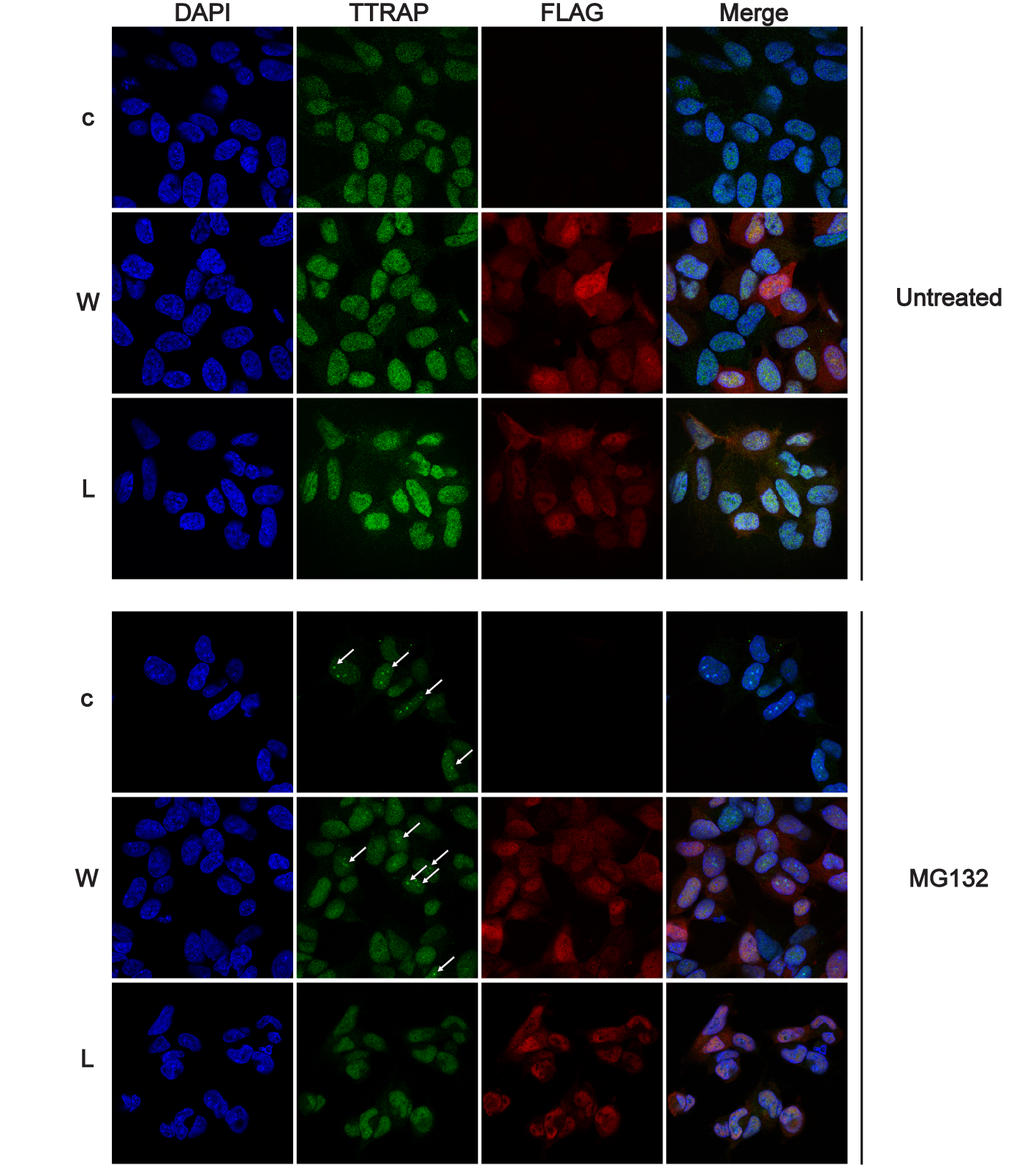

Supplement: Figure S2 — Altered TTRAP nucleolar localization in L166P mutant cells upon treatment with MG132. SH-SY5Y cells stably transfected with empty vector (c), FLAG-DJ-1 wt (W) or L166P (L) were treated with 5 µM MG132 for 16 h or left untreated. TTRAP localization was analyzed by immunofluorescence with anti-TTRAP (green) and anti-FLAG (red) antibodies. Nuclei were visualized by DAPI staining (blue). Low magnification images are shown. Nucleolar TTRAP is evident in DAPI-negative regions of the nucleus (white arrows). Images are representatives of three independent experiments from two independent clones for each cell line. (TIF) [file pone.0035051.s002.tif]

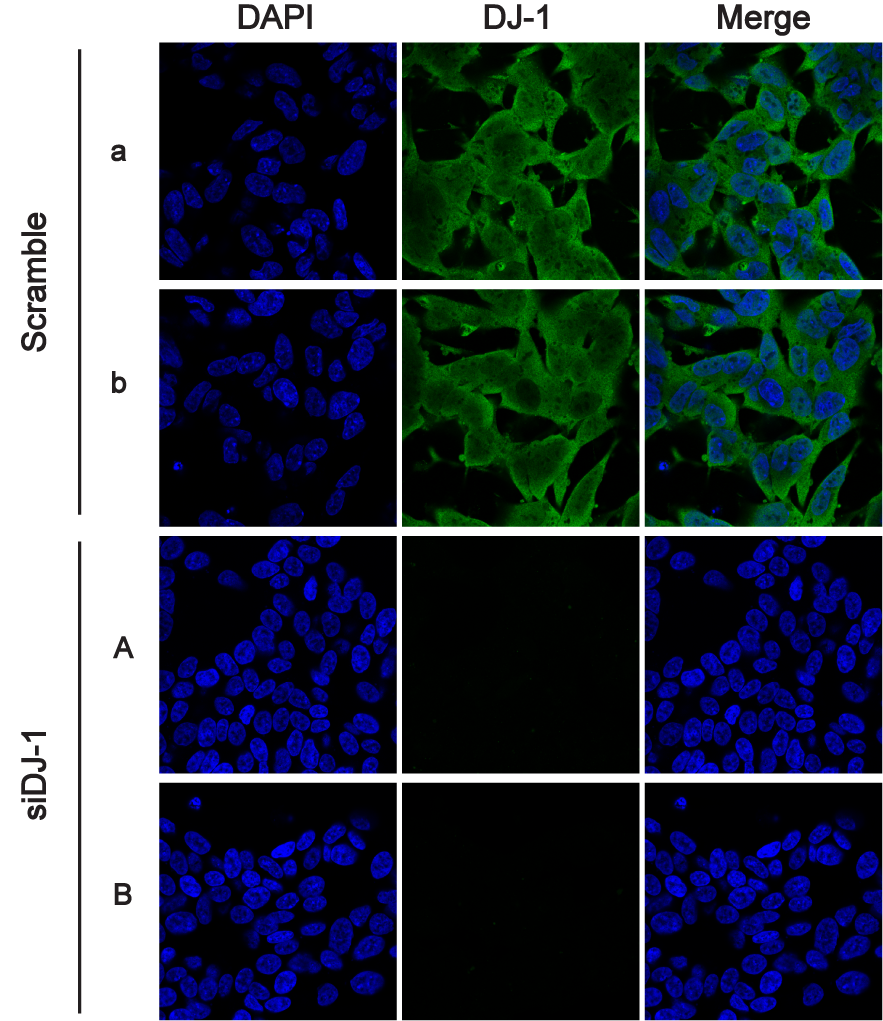

Supplement: Figure S3 — Analysis of DJ-1 expression in siDJ-1 and scramble SH-SY5Y cells. SH-SY5Y cells stably expressing a doxycyclin-inducible short-hairpin targeting DJ-1 (siDJ-1, clones A and B) or a scramble shRNA control (scramble, a and b) were treated with doxycycline for 10 days. Endogenous DJ-1 expression was analyzed by immunofluorescence with anti-DJ-1 antibody. (TIF) [file pone.0035051.s003.tif]

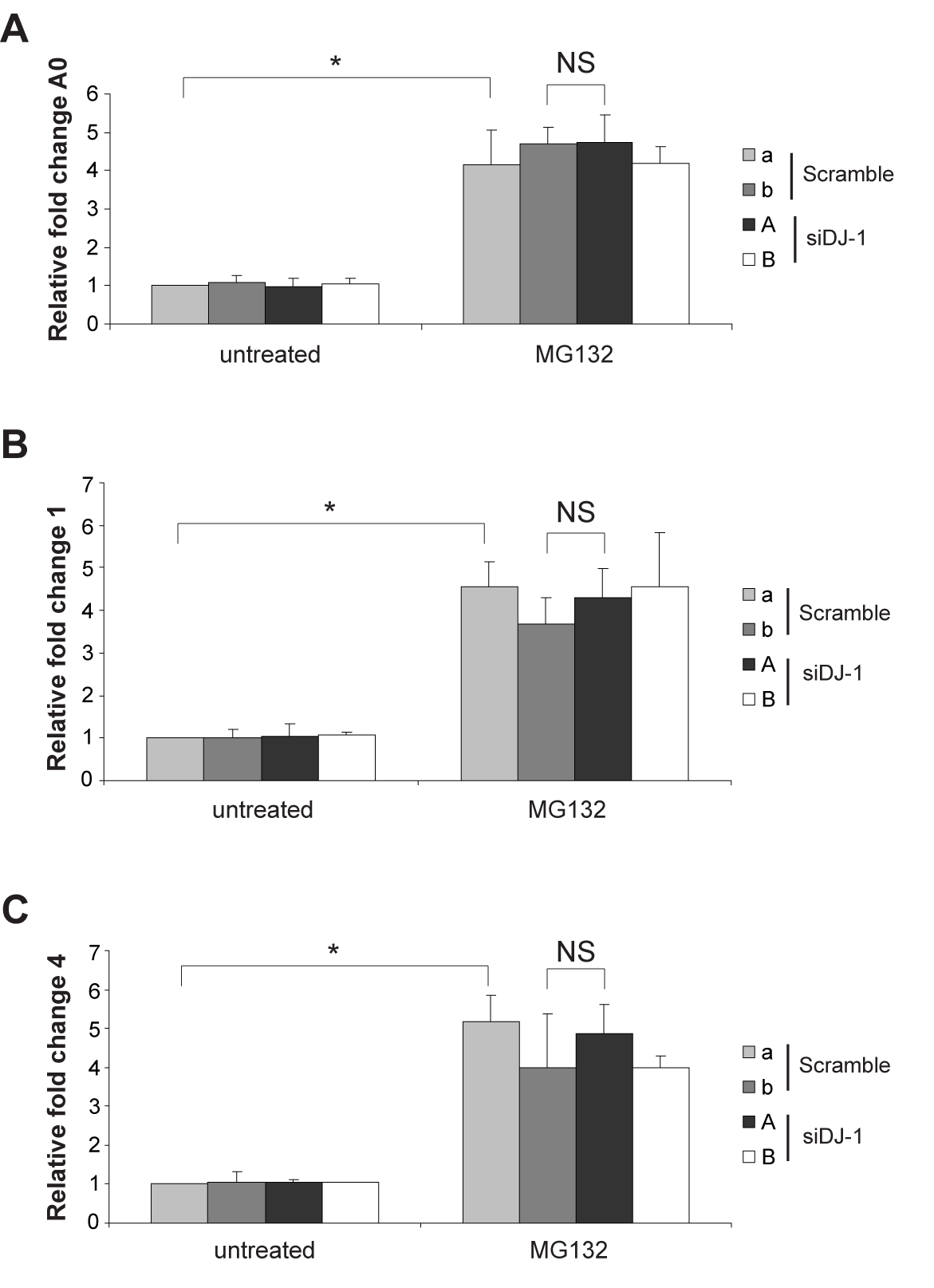

Supplement: Figure S4 — Depletion of DJ-1 does not alter transcription and processing of ribosomal RNA. SH-SY5Y stably expressing a doxycyclin-inducible shRNA targeting DJ-1 (siDJ-1, clones A and B) or a scramble shRNA control (scramble, a and b) were induced 10 days with doxyciclin and then treated with 5 µM MG132 for 16 h, or left untreated. Total RNA was extracted and levels of pre-rRNA and processing intermediates were analyzed by qPCR. Amplicons are those described in figure 3. Standard deviations are calculated from two independent experiments. Differences between a, b, A and B are not statistically significant (*, P<0.05. NS, not significant). (TIF) [file pone.0035051.s004.tif]

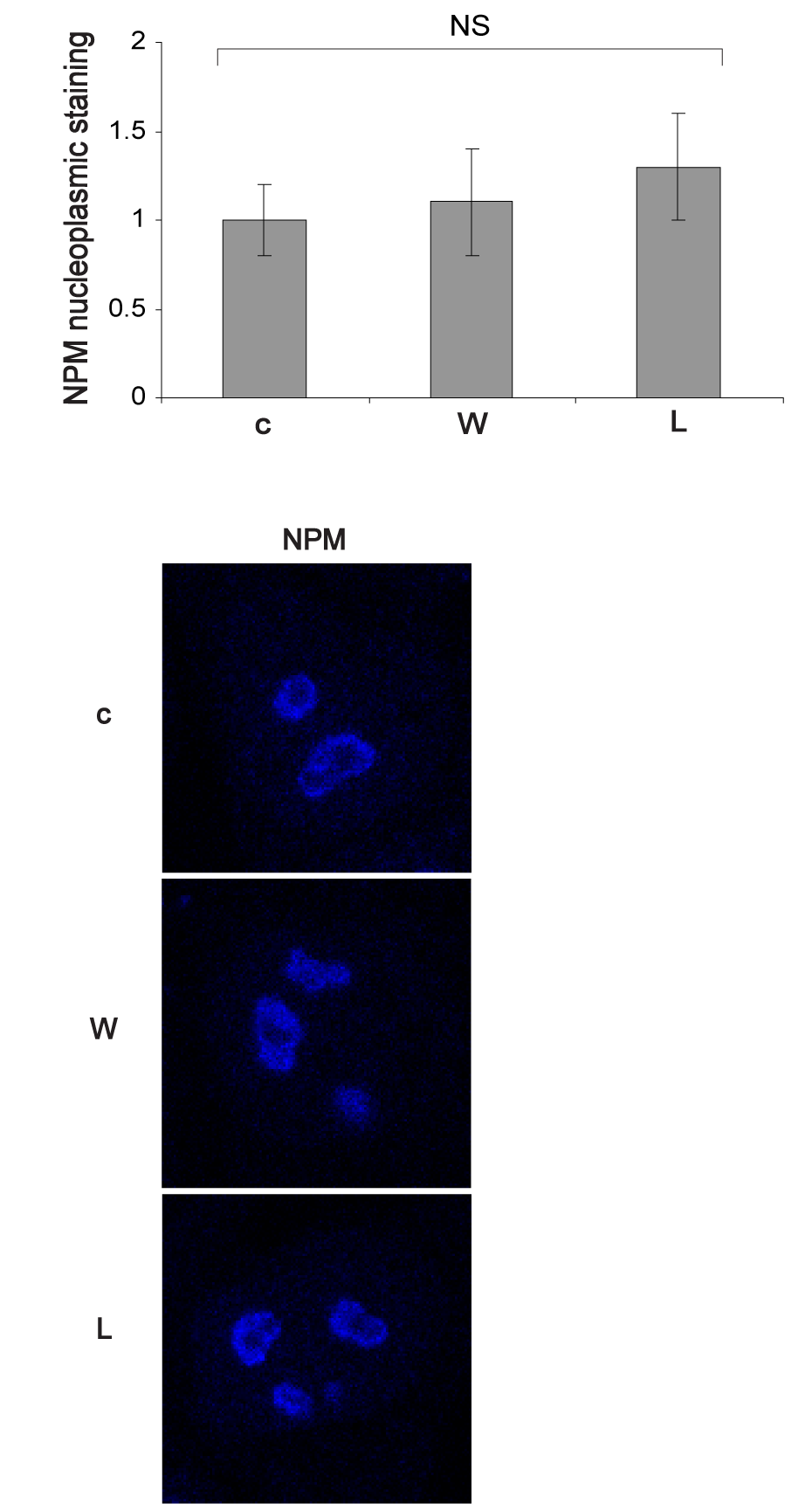

Supplement: Figure S5 — Analysis of the effects of wild-type and mutant DJ-1 on nucleolar integrity. SH-SY5Y cells stably expressing wt DJ-1 (W), L166P (L) or empty vector (c) were treated with 5 µM MG132 for 16 h. Immunofluorescence was performed with anti-NPM antibody and NPM nucleoplasmic staining was measured with ImageJ software on a randomly selected area. Background fluorescence was quantified from an area placed outside the cells and was subtracted for each signal. At least 100 cells from two separate experiments were counted (NS, not statistically significant). Representative zoomed images are shown for each cell line. (TIF) [file pone.0035051.s005.tif]

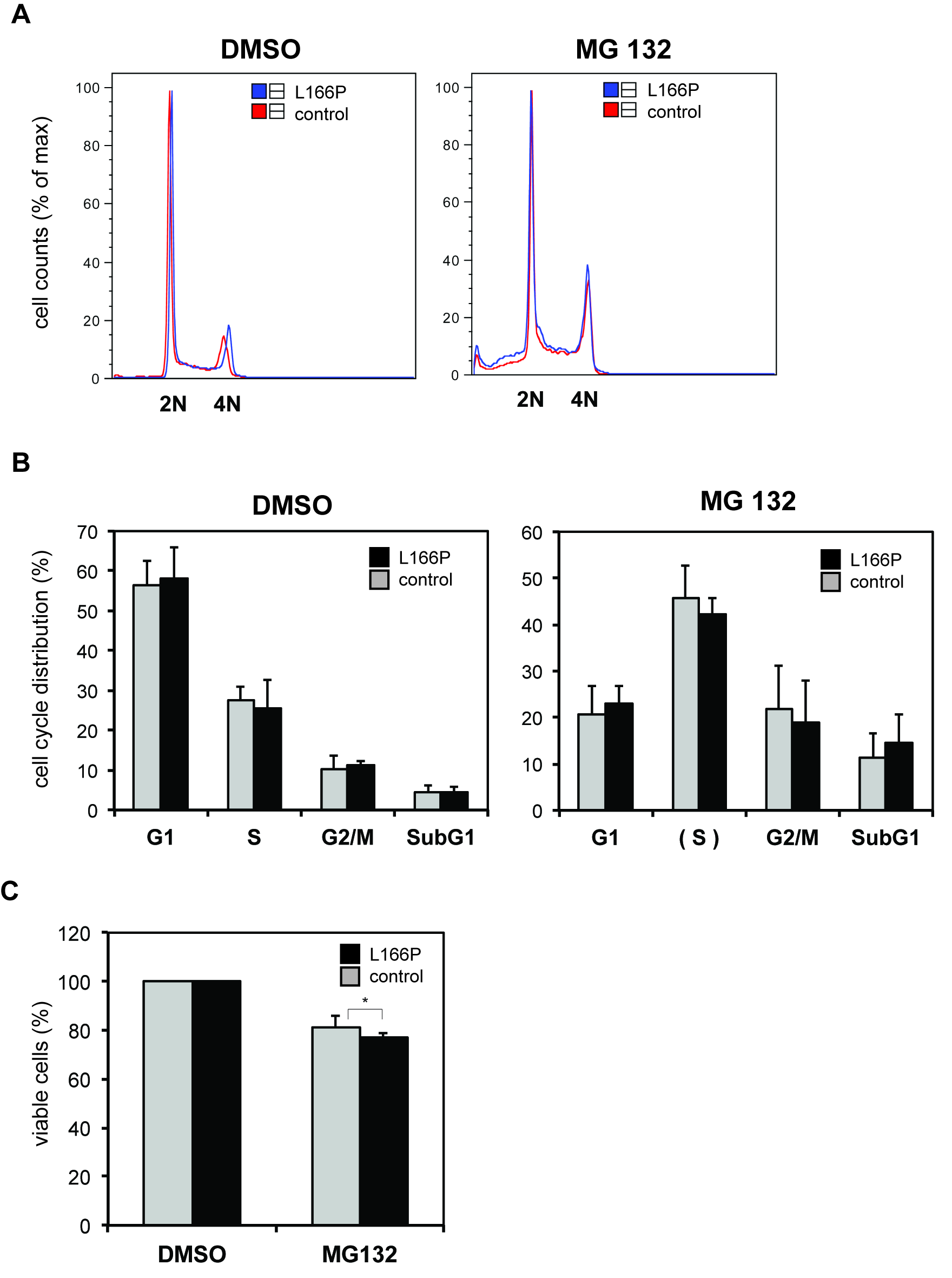

Supplement: Figure S6 — Analysis of the effects of mutant DJ-1 L166P on cell proliferation and viability. (A) Expression of L166P does not affect the cell cycle. SH-SY5Y cells stably expressing the L166P mutant and control cells were treated for 16 h with 5 µM MG132, or DMSO as control. Cell proliferation was analyzed by flow-cytometry (FACS) after propidium iodide (PI) staining. Graphs show the overlay of representative FACS profiles for each sample. (B) Quantification of the cell cycle distribution in control and L166P cells treated as in A. Data are from three independent experiments (error bars, standard deviation). (C) Expression of L166P DJ-1 moderately sensitizes cells to death induced by proteasome inhibition. Identical numbers of L166P and control cells were seeded in 96-well plates. After 24 hours, cells were treated with DMSO or 5 µM MG132 for additional 16 h. Cell viability was measured by WST-1 assay. Data are normalized to WST activity in untreated cells. Standard deviations are calculated from four independent experiments (*, p<0.05). (TIF) [file pone.0035051.s006.tif]

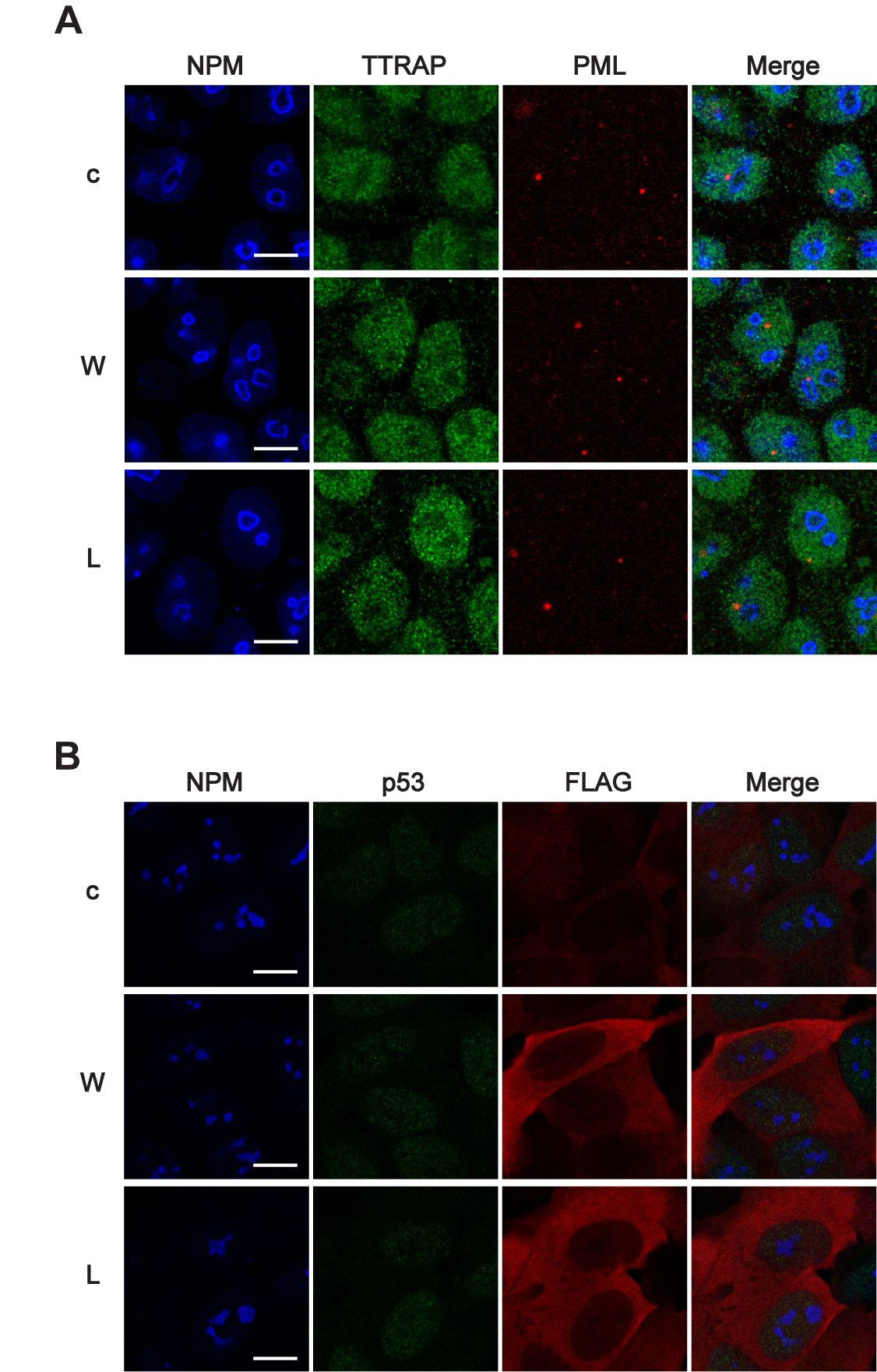

Supplement: Figure S7 — Localization of PML and p53 is not affected by the expression of DJ-1 L166P mutant in untreated cells. (A) Localization of PML. SH-SY5Y cells stably expressing FLAG-tagged DJ-1 wt (W), or mutant (L) or empty vector (c) were stained by triple immunofluorescence with anti-TTRAP (green), anti-PML (red) and anti-NPM (blue) antibodies. (B) Localization of p53. Cells were stained by triple immunofluorescence with anti-NPM (blue), anti-p53 (green) and anti-FLAG (red) antibodies. Bars, 10 µm. (TIF) [file pone.0035051.s007.tif]

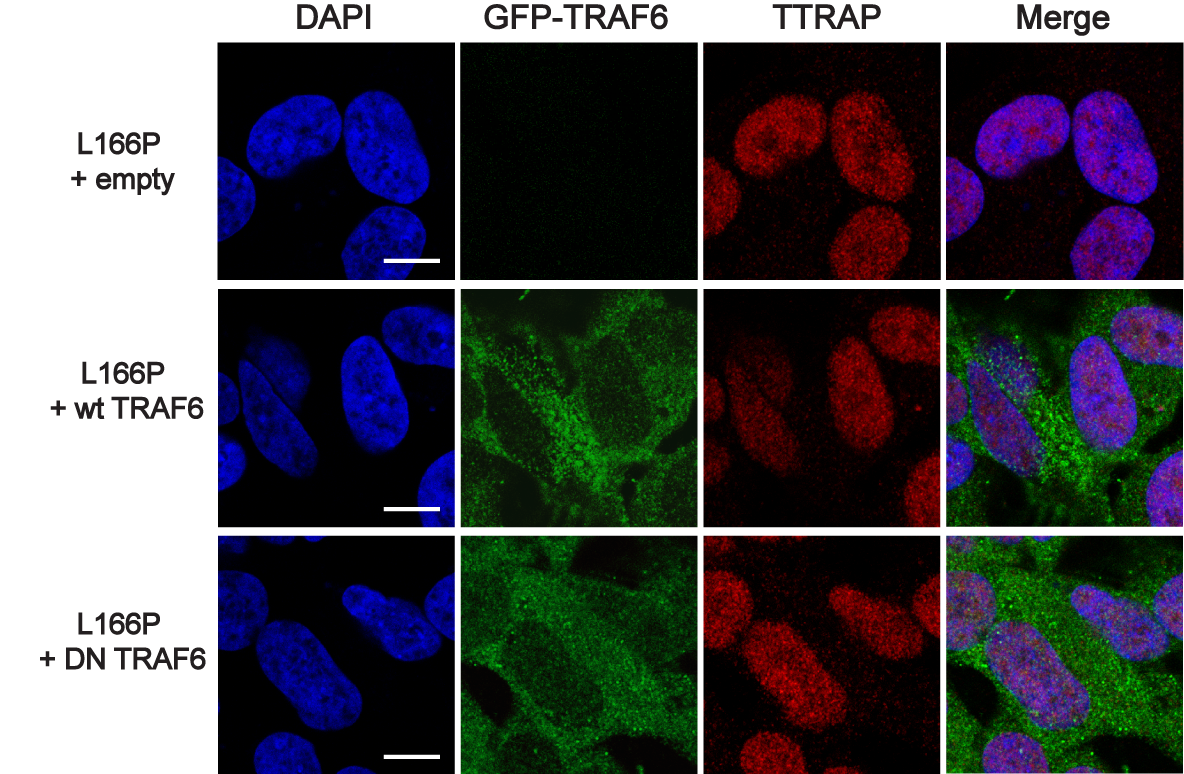

Supplement: Figure S8 — TRAF6 expression does not alter TTRAP localization in untreated cells. SH-SY5Y stably expressing L166P were transfected with GFP-TRAF6 (wt and DN), as indicated. Cells were left untreated. TTRAP localization was analyzed by immunofluorescence with anti-TTRAP (red) antibody. Bars, 10 µm. (TIF) [file pone.0035051.s008.tif]

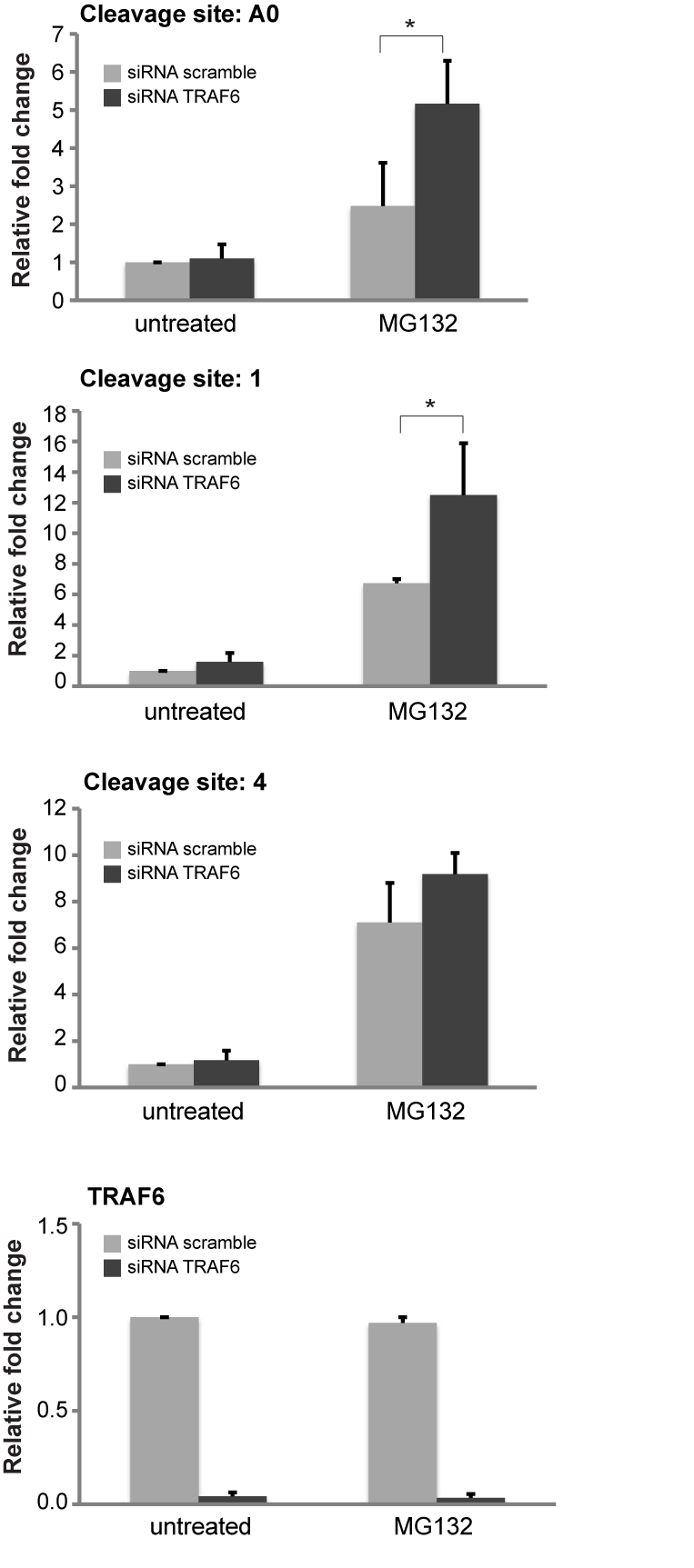

Supplement: Figure S9 — Analysis of rRNA biogenesis in mutant DJ-1 L166P cells with knock-down of TRAF6 expression. SH-SY5Y cells stably transfected with mutant DJ-1 L166P were transfected with oligonucleotides targeting endogenous TRAF6 (siRNA TRAF6) or a scramble control sequence (siRNA Scramble). After 72 h from transfection, cells were treated for 16 h with MG132 or DMSO as control. Total RNA was extracted and levels of pre-rRNA and processing intermediates were analyzed by qPCR with primers targeting A0, 1 and 4 cleavage sites, as indicated. Efficiency of TRAF6 knock-down was monitored with specific primers (TRAF6). Standard deviations are calculated from four independent experiments (*, P<0.05). (TIF) [file pone.0035051.s009.tif]
